# HEALTHCARE ENCOUNTERS AS PREDICTORS OF DEATH ANXIETY IN OLDER ADULTS

**DOI:** 10.1093/geroni/igz038.3114

**Published:** 2019-11-08

**Authors:** Todd D Becker

**Affiliations:** University of Maryland, Baltimore, Baltimore, Maryland, United States

## Abstract

Literature suggests 10–15% of Americans experience death anxiety (DA). While DA is a response to heightened death awareness, research has not fully explored the link between DA and healthcare utilization. 2012 Health and Retirement Study data were used. Three binary logistic regressions analyzed hospitalization, ER admission, nursing facility placement, and outpatient visits’ abilities to predict DA (0 = “none,” 1 = “at least some”) over the past week. Model 1 included demographics and all four types of healthcare encounters. One item from the Beck Anxiety Inventory assessed DA. Most subjects in the sample (N = 7,185) were married (n = 4,302), and White (n = 5,479). Fifteen percent reported ER admissions, 24.6% reported hospitalizations, 1.1% reported nursing facility placements, and 91.5% reported outpatient visits. Regression results from each model showed nursing facility placement and outpatient visits to be statistically significant predictors of DA across all models (OR ranges = 2.23–2.38 and 1.23–1.25 respectively, p < .05 in all models). Hospitalization predicted DA in Models 1 (OR = 1.30, p = .004) and 2 (OR = 1.46, p < .001). Model 3 showed ER admission (OR = 1.52, p < .001) predicted DA. Results from each model indicated individuals who experienced nursing facility placements exhibited double the odds of experiencing DA, compared to those who did not. Outpatient visits were the weakest predictor of DA in each model. DA’s association with healthcare utilization provides implications for providers. Future research should explore the relation between DA and health outcomes.

